# Testes de Triagem Prevendo Metástase de Câncer na Etiologia do Derrame Pericárdico: HALP Score e PNI

**DOI:** 10.36660/abc.20230376

**Published:** 2024-02-16

**Authors:** Emin Koyun, Ferhat Dindas, Anil Sahin, Idris Bugra Cerik, Mustafa Dogdus

**Affiliations:** 1 Sivas Cumhuriyet University Department of Cardiology Sivas Turquia Sivas Cumhuriyet University – Department of Cardiology, Sivas – Turquia; 2 Usak University Department of Cardiology Usak Turquia Usak University – Department of Cardiology, Usak – Turquia; 3 Ordu University Department of Cardiology Ordu Turquia Ordu University – Department of Cardiology, Ordu – Turquia; 4 Izmir University of Economics Department of Cardiology Izmir Turquia Izmir University of Economics – Department of Cardiology, Izmir – Turquia

**Keywords:** Câncer, Escore de hemoglobina, albumina, linfócito e plaqueta, Índice Prognóstico Nutricional, Efusão Pericárdica

## Abstract

**Fundamento:**

A triagem do câncer é absolutamente necessária em pacientes com derrame pericárdico, pois o câncer é uma das doenças mais graves em sua etiologia. Estudos anteriores indicaram que o índice de inflamação imunológica sistêmica (IIS), o índice prognóstico nutricional (PNI) e o escore de hemoglobina, albumina, linfócitos e plaquetas (HALP) podem ser escores relacionados ao câncer.

**Objetivos:**

Este estudo foi iniciado considerando que esses sistemas de pontuação poderiam prever o câncer na etiologia de pacientes com derrame pericárdico.

**Métodos:**

Os pacientes submetidos à pericardiocentese entre 2006 e 2022 foram analisados retrospectivamente. A pericardiocentese foi realizada em um total de 283 pacientes com derrame pericárdico ou tamponamento cardíaco de moderado a grande no período especificado. Os índices de HALP, PNI e IIS foram calculados do sangue venoso periférico retirado antes do procedimento de pericardiocentese. O nível de significância estatística foi aceito em p<0,05.

**Resultados:**

O escore HALP foi de 0,173 (0,125-0,175) em pacientes com câncer. Detectou-se que em pacientes não oncológicos o escore foi de 0,32 (0,20-0,49; p<0,001). O escore de PNI foi de 33,1±5,6 em pacientes com câncer. Detectou-se que em pacientes não oncológicos o escore foi 39,8±4,8 (p<0,001).

**Conclusão:**

Os escores HALP e PNI são testes de triagem de câncer fáceis e rápidos que podem prever metástases de câncer na etiologia de pacientes com derrame pericárdico.

**Figure:**
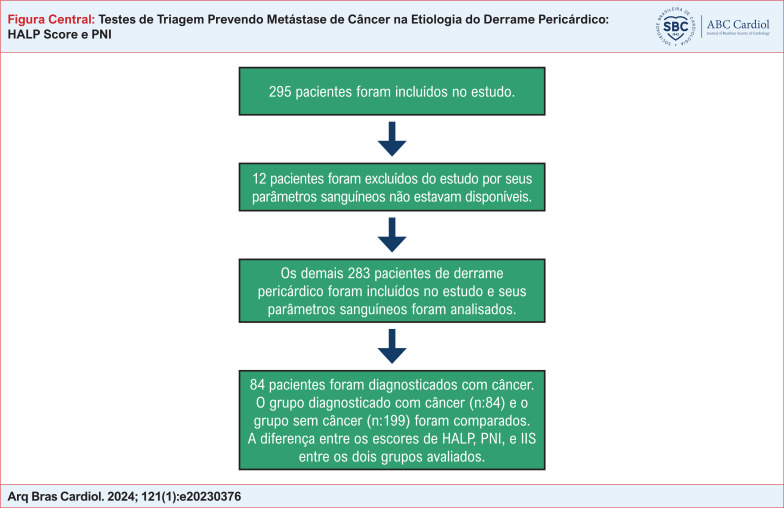


## Introdução

O acúmulo anormal de líquido no espaço pericárdico é definido como derrame pericárdico.^1^ Existem muitas causas de derrame pericárdico, como doenças infecciosas, autoimunes, neoplásicas, iatrogênicas, traumáticas, metabólicas e cardíacas.^2^

O câncer é uma doença com elevada morbidade e mortalidade. Sabe-se que a nutrição e o estado inflamatório estão entre os fatores que determinam o prognóstico em pacientes com câncer.^3^ Na literatura existem sistemas de pontuação como o escore de hemoglobina, albumina, linfócitos e plaquetas (HALP), I o índice de inflamação imunológica sistêmica (IIS) e o índice prognóstico nutricional (PNI), que estão associados a condições nutricionais e inflamatórias e, portanto, podem ser preditivos de câncer. O escore HALP, que consiste em quatro marcadores laboratoriais (hemoglobina, albumina, linfócitos e plaquetas), está associado tanto à nutrição quanto ao estado inflamatório, e é comumente usado como fator prognóstico em pacientes com vários tumores malignos, incluindo câncer gastrointestinal^4^ e câncer geniturinário.^5^

A interação entre a inflamação sistêmica, que é uma das características do câncer, e a resposta imune local desempenha um papel na formação de vários tipos de malignidades e no curso do câncer.^6,7^ Parâmetros de inflamação sistêmica também têm sido aceitos como fatores prognósticos em tumores sólidos malignos.^8^ O IIS, que consiste na contagem de linfócitos, neutrófilos e plaquetas, tem sido relatado como um fator prognóstico para pacientes com alguns tumores malignos.^9^ No câncer colorretal^10^ e esofágico,^11^ a nutrição dos pacientes e os parâmetros inflamatórios no sangue demonstraram ser eficazes na previsão do prognóstico para esses pacientes.

O PNI foi usado pela primeira vez para avaliar o risco do paciente em cirurgia para doenças gastrointestinais.^12^ O PNI, que é um sistema de pontuação que consiste na combinação do nível de albumina no soro sanguíneo e na contagem de linfócitos no sangue, é usado para avaliar o estado nutricional e imunológico em pacientes com câncer.^13^ O PNI é um escore prognóstico para carcinoma de esôfago e osteossarcoma.^14,15^

Nosso objetivo mais importante neste estudo é detectar rapidamente metástases de câncer na etiologia em pacientes com derrame pericárdico, porque a doença mais grave na etiologia dos pacientes com derrame pericárdico é o câncer. Ao investigar a etiologia em pacientes que apresentam derrame pericárdico, partimos da hipótese de que esses sistemas de pontuação, que podem ser calculados com exames laboratoriais simples, podem prever o desenvolvimento de derrame pericárdico relacionado ao câncer. Esses sistemas de pontuação são simples e de fácil aplicação. Se um sistema de pontuação para derrame pericárdico for eficaz na identificação de pacientes com câncer, ele pode acelerar o diagnóstico e o tratamento do câncer e reduzir a morbidade e a mortalidade relacionadas ao câncer.

## Métodos

Este estudo produziu uma análise retrospectiva de pacientes submetidos à pericardiocentese entre 2006 e 2022. Foi obtida a aprovação do Comitê de ética da universidade local. O número do parecer do comitê de ética foi 2022-10/11. Os pacientes e seus familiares deram o consentimento para participar do estudo. Durante o período mencionado, um total de 295 pacientes com derrame pericárdico de médio a grande porte ou tamponamento cardíaco foram submetidos à pericardiocentese com orientação de fluoroscopia (Figura Central). O tamanho do derrame pericárdico dos pacientes incluídos no estudo foi classificado de acordo com a ecocardiografia como leve (<10 mm), moderado (10-20 mm) ou grande (>20 mm) de acordo com o sistema de classificação.^16^ Todos os pacientes do presente estudo eram pacientes com derrame ou tamponamento cardíaco moderado a grande, classificados de acordo com o tamanho. Os procedimentos de pericardiocentese percutânea foram realizados principalmente no espaço subxifoide e raramente no espaço intercostal. Os registros médicos de cada paciente foram analisados para obter dados demográficos, dados clínicos e diagnósticos de doenças. Foram registrados dados do líquido pericárdico, parâmetros laboratoriais, resultados patológicos e microbiológicos no momento da admissão. O derrame foi classificado como maligno ou benigno. Se a citologia do líquido pericárdico mostrasse células malignas e suspeitas, o derrame era classificado como maligno. Aqueles sem achados anormais na citologia do derrame pericárdico foram classificados como benignos. Posteriormente, os pacientes foram divididos em dois grupos: aqueles com diagnóstico de câncer e aqueles sem diagnóstico de câncer. Foram determinados como critérios de exclusão: ter idade inferior a 18 anos, ter derrame pericárdico menor que 10 mm e ter taxa de filtração glomerular (TFG) <30 ml/min/m^2^. O diagnóstico de pericardiocentese foi feito com aparelho de ecocardiografia transtorácica. Todos os pacientes foram acompanhados com ecocardiografia transtorácica antes e após a pericardiocentese. Exames de sangue venoso periférico foram realizados em todos os pacientes com diagnóstico de derrame pericárdico. Parâmetros bioquímicos (lipoproteína de alta densidade (HDL), lipoproteína de baixa densidade (LDL), proteína C reativa (PCR), creatinina, eletrólitos séricos) foram estudados a partir das amostras de sangue coletadas. O escore IIS foi calculado com a fórmula Neutrófilos 10^3^/uL x Plaquetas 10^3^/uL / Linfócitos 10^3^/uL, o escore PNI foi calculado com a fórmula (nível sérico de albumina g/dL × 10) + (linfócitos 10^3^/uL × 0,005), e o escore HALP foi calculado com a fórmula Hemoglobina g/dL x Albumina g/dL x Linfócitos 10^3^/uL / Plaquetas 10^3^/uL.

### Análise estatística

Foram utilizados histograma, gráfico Q-Q e teste de Shapiro-Wilk para avaliar se os dados violavam as suposições de normalidade. O teste T para duas amostras e o teste U de Mann Whitney foram realizados para comparar as variáveis quantitativas entre os grupos. A análise do qui-quadrado foi utilizada para avaliar a relação entre variáveis categóricas. Os dados contínuos foram apresentados como média ± desvio padrão (DP) ou mediana (1° quartil – 3° quartil) com base na distribuição dos dados. As variáveis categóricas foram expressas como número (n) com percentual (%). A análise de regressão logística foi utilizada para determinar os fatores de risco que afetam o status do câncer. As variáveis que foram consideradas estatisticamente significativas como resultado da análise de regressão logística foram avaliadas com análise de regressão logística múltipla. A análise de característica operacional do receptor (ROC) foi realizada para avaliar os escores HALP e PNI na previsão de câncer. A área sob a curva (AUC) e o valor de corte foram calculados para cada valor do escore. A sensibilidade e a especificidade foram calculadas para avaliar o desempenho do teste diagnóstico de cada escore. O nível de significância estatística foi aceito em p<0,05. A análise dos dados foi realizada no software estatístico SPSS 22.

## Resultados

A pericardiocentese foi realizada em 295 pacientes entre 2006 e 2022. Doze pacientes foram excluídos do estudo por falta de parâmetros sanguíneos. Os demais 283 pacientes foram incluídos no estudo. Enquanto 29 dos 84 pacientes submetidos à pericardiocentese tinham diagnóstico prévio de câncer, 55 pacientes foram diagnosticados com câncer no período de 6 meses após a pericardiocentese. A doença mais comum em pacientes oncológicos foi o câncer de pulmão, enquanto o câncer de mama ficou em segundo lugar (Tabela 1).

**Tabela 1:** Distribuição de pacientes com diagnóstico de câncer

Tipo de câncer	Número de pacientes (n: 84)(%)
Câncer de pulmão	45 (% 53,57)
Câncer de mama	11 (% 13)
Câncer gástrico	8 (% 9,52)
Leucemia	4 (% 4,76)
Câncer de próstata	4 (% 4,76)
Linfoma	3 (% 3,57)
Câncer renal	2 (% 2,38)
Câncer de colo	2 (% 2,38)
Câncer de testículo	2 (% 2,38)
Câncer de bexiga	1 (% 1,19)
Câncer de tecidos moles	1 (% 1,19)
Câncer de tireoide	1 (% 1,19)

A incidência de câncer foi maior no sexo masculino. Entre os parâmetros laboratoriais comparados, os valores de hemoglobina, albumina e linfócitos foram encontrados em níveis mais baixos em pacientes com câncer, enquanto o valor de PCR foi encontrado em níveis mais elevados. Na comparação entre os sistemas de pontuação, os escores HALP e PNI foram menores em pacientes com câncer, enquanto o valor do IIS foi maior. Não houve diferença significativa entre os dois grupos, exceto nos parâmetros sexo, hemoglobina, albumina, linfócitos, PCR, HALP, IIS e PNI (Tabela 2).

**Tabela 2:** Comparação do estado do câncer e diversas variáveis

Variáveis		Câncer		p-valor
		Não (n=199)	Sim (n=84)	
Idade (anos)		63±15,9	60,6±16	0,26
Sexo, n (%)				
	Feminino	114 (57,2)	36 (42,8)	0,028
	Masculino	85 (42,8)	48 (57,2)	
FEVE (%)		50,7±12,8	51±13,4	0,64
HT, n(%)		47 (23,6)	16 (19)	0,44
DAC, n (%)		14(7)	3 (3,6)	0,411
Hemoglobina (g/dL)		13,1±1,9	11±1,7	<0,001
RBC (10^6^/uL)		4,45±0,79	4,29±0,82	0,14
WBC (10^3^/uL)		9,3±4,3	8,6±4,7	0,2
LDL (mg/dL)		97,2±40	90,7±38,2	0,25
HDL (mg/dL)		35,9±14,8	34±14,3	0,35
DM, n (%)		33 (18,0)	8 (11,3)	0,138
Ácido úrico (mg/dL)		6,27±2,4	6,15±2,3	0,76
NUS (mg/dL)		20,4±14,6	19,7±11,7	0,672
Creatinina (mg/dL)		0,8 (0,7-1,0)	0,9 (0,7-1,0)	0,128
TFG (ml/min/m²)		79,7±21,9	83,2±17,4	0,15
BASO (10^3^/uL)		0,02 (0,01-0,04)	0,02 (0,01-0,05)	0,16
EO (10^3^/uL)		0,08 (0,01-0,18)	0,07 (0,02-0,16)	0,78
HCT (%)		38,3±5,9	37,4±7	0,271
HCM		28,3±4,7	28,3±2,6	0,91
VCM (fL)		85,8±8,10	86,8±8,1	0,61
MONO (10^3^/uL)		0,55 (0,40-0,80)	0,55 (0,38-0,80)	0,37
VPM (fL)		9,3±1,26	9,3±1,46	0,34
NEUT (10^3^/uL)		7,4±4,6	7,2±4,4	0,69
PCT (%)		0,24±0,11	0,23±0,15	0,36
PDW (%)		19,55±11,6	18,2±10,3	0,37
RDW (%)		15,5±2,6	15,5±2,6	0,37
Albumina (mg/dL)		3,92±0,45	3,3±0,56	<0,001
Linfócitos (10^3^/uL)		1,84±0,66	1,29±0,81	<0,001
Plaquetas (10^3^/uL)		234±88,7	259,7±122,00	0,095
Colesterol total		162,4±52,00	152,6±47,8	0,18
PCR (mg/dL)		24,7 (8,4-81,2)	56,0 (15,0-119,0)	0,006
HALP		0,32 (0,20-0,49)	0,173 (0,125-0,175)	<0,001
IIS		857,8 (528-1664)	1329,8 (697-2272,2)	<0,001
PNI		39,8±4,8	33,1±5,6	<0,001

Os dados estão expressos em n (%), média ± desvio padrão, mediana (1° quartil - 3° quartil). FEVE: fração de ejeção ventricular esquerda; HT: hipertensão; DAC: doença arterial coronariana; RBC: hemácias; WBC: leucócitos; LDL: lipoproteína de baixa densidade; HDL: lipoproteína de alta densidade; DM: diabetes mellitus; NUS: nitrogênio ureico no sangue; TFG: taxa de filtração glomerular; BASO: basófilos; EO: eosinófilos; HCT: hematócritos; HCM: hemoglobina corpuscular média; VCM: volume corpuscular médio; MONO: monócitos; VPM: volume plaquetário médio; NEUT: neutrófilos; PCT: procalcitonina; PDW: amplitude de distribuição de plaquetas; RDW: amplitude de distribuição de hemácias; PCR: proteína C-reativa; IIS: índice de inflamação imune sistêmica; PNI: índice prognóstico nutricional.

Na análise de regressão logística múltipla, o HALP e o PNI foram preditores independentes de metástase de câncer em pacientes com derrame pericárdico (Tabela 3).

**Tabela 3:** Avaliação de fatores de risco que podem afetar o status definitivo do câncer

Variáveis		Câncer			
		Análises univariadas		Análises multivariadas	
		Razão de chance (IC 95%)	p	Razão de chance (IC 95%)	p
Sexo, n (%)					
	Feminino	1	0,027		
	Masculino	1,788 (1,068-2,994)			
Hemoglobina (g/dL)		0,514 (0,427-0,619)	<0,001		
Albumina (mg/dL)		0,066 (0,027-0,158)	<0,001		
Linfócitos (10^3^/uL)		0,318 (0,174-0,583)	<0,001		
HALP		0,003 (0,001-0,041)	<0,001	0,006 (0,001-0,090)	<0,001
PNI		0,831 (0,773-0,894)	<0,001	0,825 (0,763-0,893)	<0,001
Idade (anos)		991 (975-1007)	0,262		
DM, n (%)		515 (227-1172)	0,114		
Creatinina (mg/dL)		848 (646-1113)	0,236		
Plaquetas (10^3^/uL)		1002 (1000-1005)	0,059		
PCR (mg/dL)		1005 (1002-1009)	0,004		
IIS		1000 (1000-1001)	<0,001		

IC: intervalo de confiança; HALP: escore de hemoglobina, albumina, linfócito e plaqueta; PNI: índice prognóstico nutricional; DM: diabetes mellitus; PCR: proteína C reativa; IIS: índice de inflamação imunológica sistêmica.

**Figura 1:**
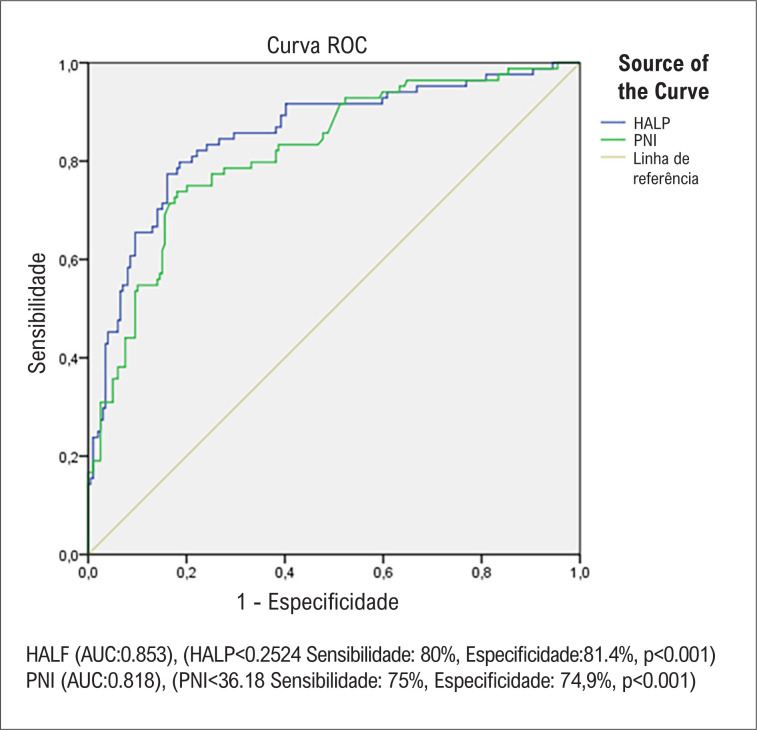
Gráficos ROC dos escores HALP e PNI. HALP: escore de hemoglobina, albumina, linfócito e plaqueta; PNI: índice prognóstico nutricional.

A análise ROC foi realizada para encontrar os valores de corte ideais de HALP e PNI para prever metástase de câncer em pacientes com derrame pericárdico. Um valor HALP <0,2524 tem sensibilidade de 80% e especificidade de 81,4% para prever metástases de câncer em pacientes com derrame pericárdico. Um valor de PNI <36,18 tem sensibilidade de 74% e especificidade de 74,9% na previsão de metástase de câncer em pacientes com derrame pericárdico. A AUC do HALP foi maior que a AUC do PNI na previsão de metástase de câncer em pacientes com derrame pericárdico (Figura 1).

## Discussão

Até onde sabemos, este é o primeiro estudo a comparar os escores HALP, IIS e PNI na pesquisa do câncer em pacientes com derrame pericárdico submetidos à pericardiocentese e a observar o efeito desses sistemas de pontuação no desenvolvimento do câncer. Neste estudo, descobriu-se que o HALP tem maior poder preditivo de metástase de câncer do que o PNI e o IIS. O HALP pode ser usado para prever o câncer em pacientes submetidos a pesquisas sobre câncer, porque é um biomarcador fácil, rápido e eficiente. O escore HALP em cânceres gastrointestinais, incluindo câncer gástrico,^17^ câncer de células escamosas de esôfago,^18^ câncer colorretal avançado,^4^ bem como em cânceres geniturinários, incluindo câncer de bexiga,^5^ carcinoma de células renais,^19^ demonstrou ter um papel prognóstico. É também um índice muito abrangente que mostra o estado nutricional e imunológico dos pacientes. Estudos anteriores demonstraram que um alto escore HALP em outros tumores prediz bons desfechos terapêuticos e prognósticos.^4,17,19^

Os escores HALP e PNI têm desempenho diagnóstico bastante semelhante na determinação do status de metástase do câncer, mas o escore HALP parece ser mais forte que o PNI. Muitos estudos mostraram que o PNI desempenha um papel no prognóstico do câncer.^13^ A razão mais importante pela qual o PNI pode apresentar uma previsão confiável para o prognóstico em pacientes com câncer é que os linfócitos ajudam o sistema imunológico e impedem a proliferação e a metástase nas células cancerígenas.^13^ A albumina sérica, outro componente do PNI, pode prever o prognóstico ao refletir o estado nutricional do organismo, fator determinante nas reações imunológicas das células cancerígenas.^13^ Em muitos estudos, o PNI de baixo nível tem sido associado a desfechos como câncer com características tumorais negativas no câncer de pulmão, câncer pouco diferenciado, câncer de grande porte e metástase.^20^ Demonstrou-se que, quanto menor o nível de PNI, mais agressivo e pior o prognóstico do câncer de pulmão.^21^ Em nosso estudo, o nível de PNI foi baixo em pacientes com metástase de câncer.

Entre os fatores mais importantes na via de inflamação do câncer estão as quimiocinas e citocinas, pequenas proteínas inflamatórias que possibilitam a comunicação intracelular no microambiente tumoral. A conexão e a comunicação entre células são muito importantes para invasão, angiogênese, crescimento tumoral e metástase. Além disso, a necrose tumoral e a ativação do fator de transcrição mediada por citocinas têm papéis importantes.^6^ O processo inflamatório no derrame pericárdico ocorre independentemente do processo patológico. Consequentemente, a produção de fluido na área pericárdica aumenta. O IIS nos mostra o equilíbrio entre o inflamatório e o imune.^22^ O IIS revelou-se um índice promissor para carcinoma hepatocelular, câncer gástrico, câncer do pulmão de pequenas células e câncer de próstata.^23^ Um alto nível de IIS indica alterações que favorecem o início, a progressão e a metástase do câncer em pacientes oncológicos.^8^ Isso pode ser explicado pelo fato de os níveis de IIS do grupo dos pacientes com câncer serem mais elevados do que os pacientes do grupo sem câncer.

Em nosso estudo, o câncer mais comum que causou derrame pericárdico foi o câncer de pulmão, seguido pelo câncer de mama e o de estômago. Em todo o mundo, o câncer de próstata e colorretal em homens e o câncer de mama e colorretal em mulheres são comuns, assim como é o câncer de pulmão. Porém, observou-se que esses tipos de câncer não causavam tanto derrame pericárdico quanto o câncer de pulmão. Isso pode estar relacionado ao fato de o câncer de pulmão metastatizar mais para o pericárdio.^21^ Além disso, neste estudo, a proporção do sexo masculino foi maior entre os pacientes com câncer. De acordo com o relatório Global Cancer Statistics, a incidência e as taxas de mortalidade por câncer de pulmão são aproximadamente 2 vezes maiores em homens do que em mulheres.^21^ Conforme os dados obtidos do Projeto Mapa do Câncer de Pulmão da Turquia, 90,4% dos pacientes com câncer de pulmão são do sexo masculino.^24^ O fato de o câncer de pulmão ser mais comum em homens pode explicar o alto índice de câncer em homens em nosso estudo. Além disso, o baixo número de pessoas diagnosticadas com câncer pode ter feito com que nossos resultados fossem assim. Portanto, são necessários grandes estudos com altos números de pacientes.

A anemia, que está entre as comorbidades relacionadas ao câncer, é frequentemente observada no momento do diagnóstico^25^ e geralmente é causada por inflamação crônica associada ao câncer.^26^ Em nosso estudo, o baixo nível de hemoglobina em pacientes diagnosticado com câncer pode ser explicado por inflamação crônica. Entretanto, como a etiologia da anemia em pacientes oncológicos não foi investigado em detalhes, os casos de hemoglobina baixa ainda não foram bem esclarecidos.

Em pacientes com câncer, a hipoalbuminemia tem sido associada a uma reação inflamatória sistêmica e à desnutrição dos pacientes.^27^ Níveis aumentados de PCR têm sido associados à diminuição da resposta dos linfócitos T às células cancerígenas.^28^ Neste estudo, os valores altos de PCR e baixos de albumina em pacientes oncológicos podem ser explicados dessa forma.

Estudos recentes mostram-nos que uma resposta inflamatória sistêmica pode desempenhar um papel importante no desenvolvimento e progressão do câncer.^29^ A inflamação sistêmica relacionada ao câncer causa linfopenia nos pacientes.^30^ É assim que podemos explicar os baixos níveis de linfócitos nos pacientes com câncer no presente estudo.

### Limitações

Este estudo tem várias limitações. É um estudo retrospectivo. Portanto, não seria possível comentar o prognóstico porque os pacientes não foram acompanhados. São necessários mais estudos multicêntricos em larga escala com acompanhamento para definir o papel do HALP e do PNI na fisiopatologia do câncer. Embora este estudo tenha identificado a associação entre esses escores e derrame pericárdico maligno, esses escores têm como objetivo determinar o prognóstico naqueles que já foram diagnosticados com câncer. Esses scores podem ser elevados em qualquer doença crônica, e isso pode levar a resultados enganosos.

## Conclusão

O HALP e o PNI são sistemas de pontuação com alto poder preditivo de metástases de câncer. Esses sistemas de pontuação são testes fáceis, rápidos e eficazes que podem ser usados na triagem de câncer.
