# Supplementary material for: Testes de Triagem Prevendo Metástase de Câncer na Etiologia do Derrame Pericárdico: HALP Score e PNI
Source: Arq Bras Cardiol. 2024 Feb 16;121(1):e20230376. [Article in Portuguese] doi: 10.36660/abc.20230376 (PMC11081201; doi:10.36660/abc.20230376)
Supplement: Translation

# Translation: Screening Tests Predicting Cancer Metastasis in the Etiology of Pericardial Effusion: HALP Score and PNI

**Authors:** Emin Koyun, Ferhat Dindas, Anil Sahin, Idris Bugra Cerik, Mustafa Dogdus

**Affiliations:** 1 Sivas Turkey Sivas Cumhuriyet University – Department of Cardiology, Sivas – Turkey; 2 Usak Turkey Usak University – Department of Cardiology, Usak – Turkey; 3 Ordu Turkey Ordu University – Department of Cardiology, Ordu – Turkey; 4 Izmir Turkey Izmir University of Economics – Department of Cardiology, Izmir – Turkey

**Keywords:** Cancer, Hemoglobin, Albumin, Lymphocyte, Platelet (HALP), Prognostic Nutrition İndex, Pericardial Effusion

## Abstract

**Background:**

Cancer screening is absolutely necessary in patients with pericardial effusion, given that cancer is one of the most serious diseases in the etiology of pericardial effusion. In previous studies, it was stated that the systemic immune-inflammation index (SII); the prognostic nutrition index (PNI); and the hemoglobin, albumin, lymphocyte, platelet (HALP) score can produce scores related to cancer.

**Objectives:**

This study began considering that these scoring systems could predict cancer in the etiology of patients with pericardial effusion.

**Methods:**

This study produced a retrospective analysis of patients who underwent pericardiocentesis between 2006 and 2022. Pericardiocentesis was performed in a total of 283 patients with moderate-to-large pericardial effusion or pericardial tamponade within the specified period. HALP, PNI, and SII scores were calculated according to the peripheral venous blood taken before the pericardiocentesis procedure. The statistical significance level was set at p<0.05.

**Results:**

The HALP score proved to be 0.173 (0.125-0.175) in cancer patients and 0.32 (0.20-0.49) in non-cancer patients (p<0.001). The PNI score proved to be 33.1±5.6 in cancer patients and 39.8±4.8 in non-cancer patients (p<0.001).

**Conclusion:**

The HALP score and PNI proved to be easy and fast cancer screening tests that can predict cancer metastasis in the etiology of patients with pericardial effusion.

## Introduction

Abnormal fluid accumulation in the pericardial space is defined as pericardial effusion.^1^ There are many causes of pericardial effusion, such as infections and autoimmune, neoplastic, iatrogenic, traumatic, metabolic diseases, and heart diseases.^2^

Cancer is a disease with a high morbidity and mortality. It is also well-known that nutrition and inflammatory status are among the factors determining the prognosis in cancer patients.^3^ In the literature, scoring systems, such as the hemoglobin, albumin, lymphocyte, platelet (HALP), the systemic immune-inflammation index (SII), and the prognostic nutrition index (PNI) scores are associated with nutritional and inflammatory conditions and can thus be predictive of cancer. The HALP score, which consists of four laboratory markers (hemoglobin, albumin, lymphocyte, platelet), is associated with both nutrition and inflammatory status, and is commonly used as a prognostic factor in patients with various malignant tumors, including gastrointestinal cancer^4^ and genitourinary cancer.^5^

The interaction between systemic inflammation, which is one of the features of cancer, and local immune response plays a role in the formation of various types of malignancy and in the course of cancer.^6,7^ Systemic inflammation parameters have also been accepted as prognostic factors in malignant solid tumors.^8^ SII, which consists of lymphocyte, neutrophil, and platelet counts, has been reported to be a prognostic factor for patients with some malignant tumors.^9^ In colorectal^10^ and esophageal cancer,^11^ nutrition of patients and inflammatory parameters in the blood have proven to be effective in predicting prognoses in these patients.

PNI, by contrast, was first used to assess patient risk in surgery for gastrointestinal diseases.^12^ PNI, which is a scoring system consisting of the combination of the albumin level in blood serum and lymphocyte counts in the blood, is used to evaluate nutritional and immune statuses in cancer patients,^13^ and serves as a prognostic score in esophageal carcinoma and osteosarcoma.^14,15^

Our most important aim in this study is to quickly detect cancer metastasis in etiology in patients with pericardial effusion. Because the most serious disease in the etiology of patients with pericardial effusion is cancer. While investigating the etiology in patients presenting pericardial effusion, we first establihsed the hypothesis that these scoring systems, which can be calculated with simple laboratory tests, can predict the development of cancer-related pericardial effusion. These scoring systems are simple and easy to apply. If a scoring system for pericardial effusion is found to be effective in identifying patients with cancer, these scoring systems can accelerate the diagnosis and treatment of cancer and reduce cancer-related morbidity and mortality.

## Methods

This study produced a retrospective analysis of patients who underwent pericardiocentesis between 2006 and 2022. Approval from the ethics committee of the local university was obtained, logged under ethics committee decision number: 2022-10/11. Consent was obtained from the patients and their relatives to participate in the study. During the stated period, a total of 295 patients with medium to large pericardial effusion or pericardial tamponade underwent pericardiocentesis under fluoroscopy guidance (Central Illustration). Pericardial effusion size of the patients from the present study was classified according to the echocardiography as mild (<10 mm), moderate (10–20 mm), or large (>20 mm) according to the classification system.^16^ All of the patients included in our study were patients with moderate-to-large effusion or cardiac tamponade, classified according to size. Percutaneous pericardiocentesis procedures were performed mostly from the subxiphoid space and rarely from the intercostal space. Each patient's medical records were reviewed to obtain patient demographics, clinical data, and disease diagnoses. Pericardial fluid data, laboratory parameters, pathology results and microbiology results at the time of admission were recorded. The effusion was classified as either malignant or benign. If the pericardial fluid cytology showed malignant and suspicious cells, the effusion was classified as malignant. Those without abnormal findings in pericardial effusion cytology were classified as benign. The patients were then divided into two groups: those diagnosed with cancer and those not diagnosed with cancer. The following were determined as exclusion criteria: being younger than 18 years of age, having a pericardial effusion less than 10 mm, and having a glomerular filtration rate (GFR) <30 ml/min/m^2^. The diagnosis of pericardiocentesis was conducted using the transthorasic echocardiogram (TTE). All patients underwent follow-up via TTE before and after pericardiocentesis. Peripheral venous blood tests were taken from all patients diagnosed with pericardial effusion. Biochemistry parameters (high density lipoprotein (HDL), low density lipoprotein (LDL), C-reactive protein (CRP), creatinine, serum electrolytes) were analyzed according to the blood samples. The SII score was calculated using the Neutrophil 10^3^/uL x Platelet 10^3^/uL / Lymphocyte 10^3^/uL formula, while the PNI score was calculated using the (serum albümin g/dL level × 10) + (lymphocyte 10^3^/uL × 0.005) formula. The HALP score, by contrast, was calculated using the Hemoglobin g/dL x Albumin g/dL x Lymphocyte 10^3^/uL / Platelet 10^3^/uL formula.

### Statistical analysis

Histogram, q-q graph and Shapiro-Wilk test were used to evaluate whether or not the data violated normality assumptions. The T-test and Mann Whitney U test were performed on two-samples in order to compare quantative variables between groups. The chi-square analysis was used to evaluate the relationship between categorical variables. The continuous data were presented as mean ± standard deviation (SD) or median (1^st^ quartile – 3d quartile) based on the data distribution. The categorical variables were expressed as the number (n) with a percentage (%). Logistic regression analysis was used to determine the risk factors affecting the cancer status. Variables that were found to be statistically significant as a result of logistic regression analysis were evaluated using multiple logistic regression analysis. Receiver operating characteristic (ROC) analysis was performed to evaluate the HALP and PNI scores used to predict cancer. The area under the curve (AUC) and the cut-off value were calculated for each score value. Sensitivity and specificity were calculated to evaluate the diagnostic test performance of each score. The statistical significance level was set at p<0.05. Data analysis was performed using SPSS 22 statistical software.

## Results

Pericardiocentesis was performed on 295 patients between 2006 and 2022. Twelve patients were excluded from the study due to a lack of blood parameters. The remaining 283 patients were included in the study. While 29 of 84 patients who underwent pericardiocentesis had a previous diagnosis of cancer, 55 patients were diagnosed with cancer within 6 months after pericardiocentesis. The most common disease was lung cancer, with breast cancer ranking second (Table 1).

**Table 1:** Distribution of patients diagnosed with cancer

Cancer Type	Number of patients (n: 84)(%)
Lung cancer	45 (%53.57)
Breast cancer	11 (%13)
Gastric cancer	8 (%9.52)
Leukemia	4 (%4.76)
Prostate cancer	4 (%4.76)
Lymphoma	3 (%3.57)
Renal cancer	2 (%2.38)
Colon cancer	2 (%2.38)
Testis cancer	2 (%2.38)
Bladder cancer	1 (%1.19)
Soft tissue cancer	1 (%1.19)
Thyroid cancer	1 (%1.19)

The incidence of cancer was higher in the male gender. Among the compared laboratory parameters, hemoglobin, albumin, and lymphocyte values were found at lower levels in cancer patients, while the CRP value was found at higher levels. In the comparison between scoring systems, the HALP and PNI scores proved to be lower in cancer patients, while the SII value was higher. No significant difference was found between the two groups except for sex, hemoglobin, albumin, lymphocyte, CRP, HALP, SII, and PNI parameters (Table 2).

**Table 2:** Comparison of cancer status and various variables

Variables		Cancer		p-value
		No (n=199)	Yes (n=84)	
Age (years)		63±15.9	60.6±16	0.26
Sex n(%)				
	Female	114(57.2)	36(42.8)	0.028
	Male	85(42.8)	48(57.2)	
LVEF (%)		50.7±12.8	51±13.4	0.64
HT n(%)		47(23.6)	16(19)	0.44
CAD n(%)		14(7)	3(3.6)	0.411
Hemoglobin (g/dL)		13.1±1.9	11±1.7	<0.001
RBC (10^6^/uL)		4.45±0.79	4.29±0.82	0.14
WBC (10^3^/uL)		9.3±4.3	8.6±4.7	0.2
LDL (mg/dL)		97.2±40	90.7±38.2	0.25
HDL (mg/dL)		35.9±14.8	34±14.3	0.35
DM n(%)		33(18.0)	8(11.3)	0.138
Uric asid (mg/dL)		6.27±2.4	6.15±2.3	0.76
BUN (mg/dL)		20.4±14.6	19.7±11.7	0.672
Creatinine (mg/dL)		0.8(0.7-1.0)	0.9(0.7-1.0)	0.128
GFR (ml/min/m2)		79.7±21.9	83.2±17.4	0.15
BASO (10^3^/uL)		0.02(0.01-0.04)	0.02(0.01-0.05)	0.16
EO (10^3^/uL)		0.08(0.01-0.18)	0.07(0.02-0.16)	0.78
HCT (%)		38.3±5.9	37.4±7	0.271
MCH		28.3±4.7	28.3±2.6	0.91
MCV (fL)		85.8±8.10	86.8±8.1	0.61
MONO (10^3^/uL)		0.55(0.40-0.80)	0.55(0.38-0.80)	0.37
MPV (fL)		9.3±1.26	9.3±1.46	0.34
NEUT (10^3^/uL)		7.4±4.6	7.2±4.4	0.69
PCT (%)		0.24±0.11	0.23±0.15	0.36
PDW (%)		19.55±11.6	18.2±10.3	0.37
RDW (%)		15.5±2.6	15.5±2.6	0.37
Albumin (g/dL)		3.92±0.45	3.3±0.56	<0.001
Lymphocyte (10^3^/uL)		1.84±0.66	1.29±0.81	<0.001
Platelet (10^3^/uL)		234±88.7	259.7±122.00	0.095
Total cholesterol		162.4±52.00	152.6±47.8	0.18
CRP (mg/L)		24.7(8.4-81.2)	56.0(15.0-119.0)	0.006
HALP		0.32(0.20-0.49)	0.173(0.125-0.175)	<0.001
SII		857.8(528-1664)	1329.8(697-2272.2)	<0.001
PNI		39.8±4.8	33.1±5.6	<0.001

Data are expressed as n(%), mean ± standard deviation, median(1st quartile - 3rd quartile). LVEF: left ventricular ejection fraction; HT: hypertension; CAD: coronary artery disease; RBC: red blood cells; WBC: white blood cells; LDL: low density lipoprotein; HDL: high density lipoprotein; DM: diabetes mellitus; BUN: blood urea nitrogen; GFR: glomerular filtration rate; BASO: basophil; EO: eosinophil; HCT: hematocrit; MCH: mean corpuscular hemoglobin; MCV: mean corpuscular volume; Mono: monocyte; MPV: mean platelet volume; NEUT: neutrophyl; PCT: procalcitonin; PDW: platelet distribution width; RDW: redcell distribution width; CRP: C-reactive protein; SII: systemic immune-inflammation index; PNI: prognostic nutritional index.

In multiple logistic regression analysis, HALP and PNI proved to be independent predictors of cancer metastasis in patients with pericardial effusion (Table 3).

**Table 3:** Evaluation of risk factors that may affect definitive cancer status

Variables		Cancer			
		Univariate analyses		Multivariate analyses	
		Odds Ratio (95% CI)	p-value	Odds Ratio (95% CI)	p-value
Sex n(%)					
	Female	1	0.027		
	Male	1.788(1.068-2.994)			
Hemoglobin (g/dL)		0.514(0.427-0.619)	<0.001		
Albumin (g/dL)		0.066(0.027-0.158)	<0.001		
Lymphocyte (10^3^/uL)		0.318(0.174-0.583)	<0.001		
HALP		0.003(0.001-0.041)	<0.001	0.006(0.001-0.090)	<0.001
PNI		0.831(0.773-0.894)	<0.001	0.825(0.763-0.893)	<0.001
Age (years)		991(975-1007)	0.262		
DM n(%)		515(227-1172)	0.114		
Creatinine (mg/dL)		848(646-1113)	0.236		
Platelet (10^3^/uL)		1002(1000-1005)	0.059		
CRP (mg/L)		1005(1002-1009)	0.004		
SII		1000(1000-1001)	<0.001		

CI: confidence ınterval; HALP: hemoglobin-albumin-lymphocyte-platelet index; PNI: prognostic nutritional index; DM: diabetes mellitus; CRP: C-reactive protein; SII: systemic immune-inflamation index.

ROC analysis was performed to find the ideal cut-off values of HALP and PNI to predict cancer metastasis in patients with pericardial effusion. A HALP value of <0.2524 has an 80% sensitivity and an 81.4% specificity to predict cancer metastasis in patients with pericardial effusion. A PNI value of <36.18 has a sensitivity of 74% and a specificity of 74.9% in predicting cancer metastasis in patients with pericardial effusion. The AUC of HALP proved to be higher than the AUC of PNI in predicting cancer metastasis in patients with pericardial effusion (Figure 1).

**Figure 1:** ROC graphs of HALP and PNI scores. HALP: hemoglobin-albumin-lymphocyte-platelet index; PNI: prognostic nutritional index.

## Discussion

To the best of our knowledge, this is the first study to compare HALP, SII, and PNI scores in cancer research among patients with pericardial effusion who underwent pericardiocentesis, and to look at the effect of these scoring systems on cancer development. In this study, HALP proved to have a greater predictive power for cancer metastasis than did PNI and SII. HALP can be used to predict cancer in patients undergoing cancer research, considering that it is an easy, quick, and effective biomarker. The HALP score in gastrointestinal cancers, including gastric cancer,^17^ esophageal squamous cell cance,^18^ advanced colorectal cancer,^4^ as well as in genitourinary cancers, including bladder cancer^5^ and renal cell carcinoma,^19^ has proven to have a prognostic role. It is also a very comprehensive index that shows both the nutritional and immune status of patients. Previous studies have shown that a high HALP score in other tumors can predict good therapeutic outcomes and prognosis.^4,17,19^

The HALP and PNI scores have quite similar diagnostic performances in determining the status of cancer metastasis; however, the HALP score appears to be stronger than the PNI. Many studies have shown that PNI plays a role in cancer prognosis.^13^ The most important reason why PNI can provide a reliable prediction of the prognosis in cancer patients is that lymphocytes help the immune system and hinder proliferation and metastasis in cancer cells.^13^ Serum albumin, another PNI component, can predict the prognosis by reflecting the nutritional status of the body, which is a determining factor in the immune reactions of cancer cells.^13^ In many studies, low-level PNI has been associated with outcomes, including cancer with negative tumor characteristics in lung cancer, poorly differentiated cancer, large-sized cancer, and metastasis.^20^ It has been shown that a lower PNI level can predict a more aggressive cancer and a worse prognosis of lung cancer.^21^ In our study, the PNI level proved to be quite low in patients with cancer metastasis.

Among the most important factors in the cancer inflammation pathway are chemokines, cytokines, and small inflammatory proteins, all of which provide intracellular communication in the tumor microenvironment, as well as connection and communication between cells, which is very important when predicting invasion, angiogenesis, tumor growth, and metastasis. In addition, tumor necrosis and cytokine-mediated transcription factor activation also play important roles.^6^ The inflammatory process in pericardial effusion occurs depending on the pathological process. Accordingly, fluid production in the pericardial area increases. SII shows us the balance between the inflammatory and immune processes.^22^ SII proved to be a promising index for hepatocellular carcinoma, gastric cancer, small cell lung cancer, and prostate cancer.^23^ A high SII level indicates changes that favor cancer initiation, progression, and metastasis in cancer patients.^8^ This can be explained by the fact that the SII levels of the cancer patients group were higher than those of the patients in the non-cancer group.

In our study, the most common cancer causing pericardial effusion was lung cancer, followed by breast and stomach cancer. Prostate and colorectal cancer in men and breast and colorectal cancers in women are also common cancers, similar to lung cancer. However, it was observed that these types of cancer did not cause pericardial effusion as much as lung cancer did. This may be related to the fact that lung cancer metastasizes more to the pericardium.^21^ In addition, in this study, the male sex ratio proved to be higher among patients with cancer. According to the Global Cancer Statistics report, lung cancer incidence and death rates are reported to be approximately 2-fold higher in men than in women.^21^ According to data obtained from the Turkey Lung Cancer Map Project, 90.4% of all lung cancer cases are male.^24^ Since lung cancer is most common in men, the high rate of cancer in men in our study can be explained in a similar manner. In addition, the low number of people diagnosed with cancer may have to the results from our study. Therefore, larger studies with a larger numbers of patients are warranted.

Anemia, which is among the cancer-related comorbidities, is frequently seen at the time of diagnosis^25^ and is commonly caused by chronic inflammation associated with cancer.^26^ In our study, the low hemoglobin level in patients diagnosed with cancer can be explained by chronic inflammation. However, since the etiology of anemia in cancer patients has not been investigated in detail, the causes of low hemoglobin have not been fully clarified.

In cancer patients, hypoalbuminemia has been associated with a systemic inflammatory reaction and malnutrition in patients.^27^ Increased CRP levels have been associated with decreased T lymphocyte response to cancer cells.^28^ In our study, high CRP and low albumin values in cancer patients can be explained in a similar manner.

Recent studies show us that a systemic inflammatory response may play an important role in the development and progression of cancer.^29^ Cancer-related systemic inflammation causes lymphopenia in patients,^30^ which provides a logical explanation for the low lymphocyte levels in cancer patients in the present study.

### Limitations

This study has several limitations. It is a retrospective study. Therefore, the prognosis could not be commented on, as the patients did not receive follow-up. More large-scale, multicenter studies with follow-up are needed to define the role of HALP and PNI in the pathophysiology of cancer. Although this study identified an association between those scores and a malignant pericardial effusion, those scores are meant to determine prognosis in those who were already diagnosed with cancer. Those scores can be realtively high in any chronic disease, which can lead to misleading results.

## Conclusion

HALP and PNI are scoring systems with high predictive power for cancer metastasis. These scoring systems are easy, quick, and effective tests that can be used in cancer screening.
